# Neurofibromatosis type I with breast cancer: not only for women!

**DOI:** 10.1186/1897-4287-12-5

**Published:** 2014-02-24

**Authors:** Kuntegowdanahalli Chinnagiriyappa Lakshmaiah, Anil N Kumar, Samit Purohit, Belathur Kalegowda Viveka, Kamalakannan Rahul Rajan, Mohammed Abdul Lateef Zameer, Prabhu Namitha, Monika Lamba Saini, Hatem A Azim, Kamal S Saini

**Affiliations:** 1 Department of Medical Oncology, Kidwai Memorial Institute of Oncology, Bangalore 560030, India; 2 Department of Surgical Oncology, Kidwai Memorial Institute of Oncology, Bangalore, India; 3 Department of Pathology, Kidwai Memorial Institute of Oncology, Bangalore India; 4 Department of Dermatology, Bangalore Medical College and Research Institute, Bangalore India; 5 Department of Anatomie Pathologique, Université Catholique de Louvain, Brussels Belgium; 6 Department of Medical Oncology, BrEAST Data Centre, Institut Jules Bordet, Université Libre de Bruxelles, Brussels Belgium; 7 Department of Medical Oncology, Breast International Group, Institut Jules Bordet, Université Libre de Bruxelles, Brussels Belgium

## Abstract

The association of neurofibromatosis type I with invasive male breast cancer is a rare clinical entity with only one case in literature reported in 1953. Women with NF1 are at risk of developing breast cancer and men also may be at risk but there is scarce data on the risk and association of NF1 with male breast cancer due to its rarity. Established clinical trials in male breast cancer patients are lacking and the results are extrapolated from female breast cancer patients. The treatment of male breast cancer is followed as per the guidelines of premenopausal female breast cancer and tamoxifen is the hormone treatment in them. Mendes et al suggests that silencing of NF1 gene confers resistance to tamoxifen. Our conclusions are that since NF1 is mutated or deleted in one third of sporadic breast cancers, its role as a molecular driver for treatment has to be further explored.

## Introduction

Male breast cancer is a rare disease and accounts for less than 1% of all breast cancers [1]. Neurofibromatosis type 1 (NF1) or Von Recklinghausen disease is an autosomal dominant condition that leads to multiple benign tumors (neurofibromas) and predisposes to several types of malignancies, including breast cancer. To the best of our knowledge, only 1 case of neurofibromatosis in a male patient with breast cancer has been previously reported in the literature [2]. Here we report another case and discuss the potential role of the *NF1* gene in the pathogenesis of these cases.

## Case description

A 55-year-old male presented to our institution in November 2011 with complaints of multiple skin nodules, brown skin patches since childhood and an enlarging painless lump in the left breast for 2 months. There was no other medical history of relevance including food allergies or drug abuse. There was history of similar skin nodules in his brother and paternal uncle. There was no history of breast, ovary or prostate cancer in the family.

**Figure 1:**
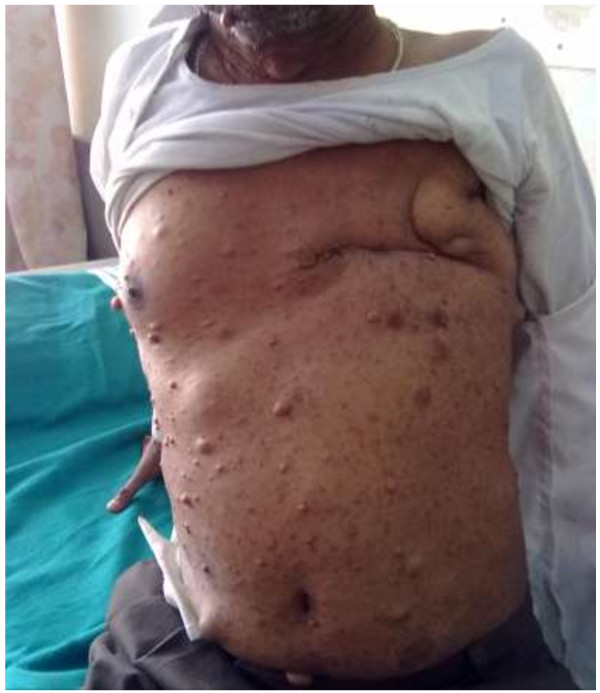
Clinical photograph of male patient with multiple sessile to pedunculated, dome shaped, skin colored nodules, multiple cafe-u-lait spots, and a left modified radical mastectomy scar.

On examination, multiple, soft, sessile to pedunculated, dome shaped, skin colored nodules (Figure 1) with size ranging from 0.5-4 cms and eight well circumscribed brown macular patches distributed all over the body, with size ranging from 0.5-6 cms, were observed. On ophthalmologic examination, the iris and retina were normal. An irregular lump in left breast of size 6 × 2 cms was noted. It was hard in consistency, non-tender, and mobile; axillary lymph nodes were not palpable. Hematological, renal and liver function tests were normal.

**Figure 2:**
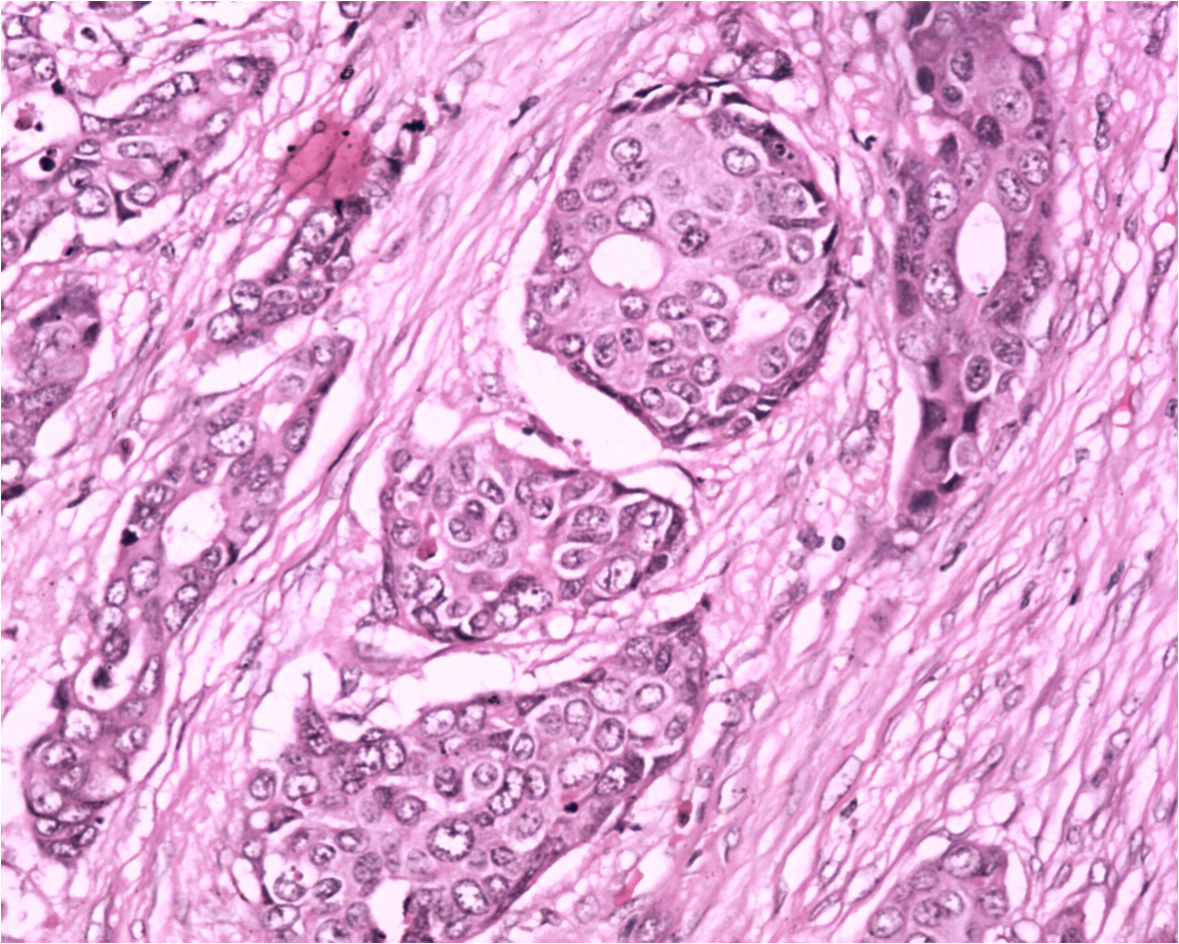
High magnification of invasive ductal carcinoma of breast with tumor nests and marked nuclear pleomorphism.

**Figure 3:**
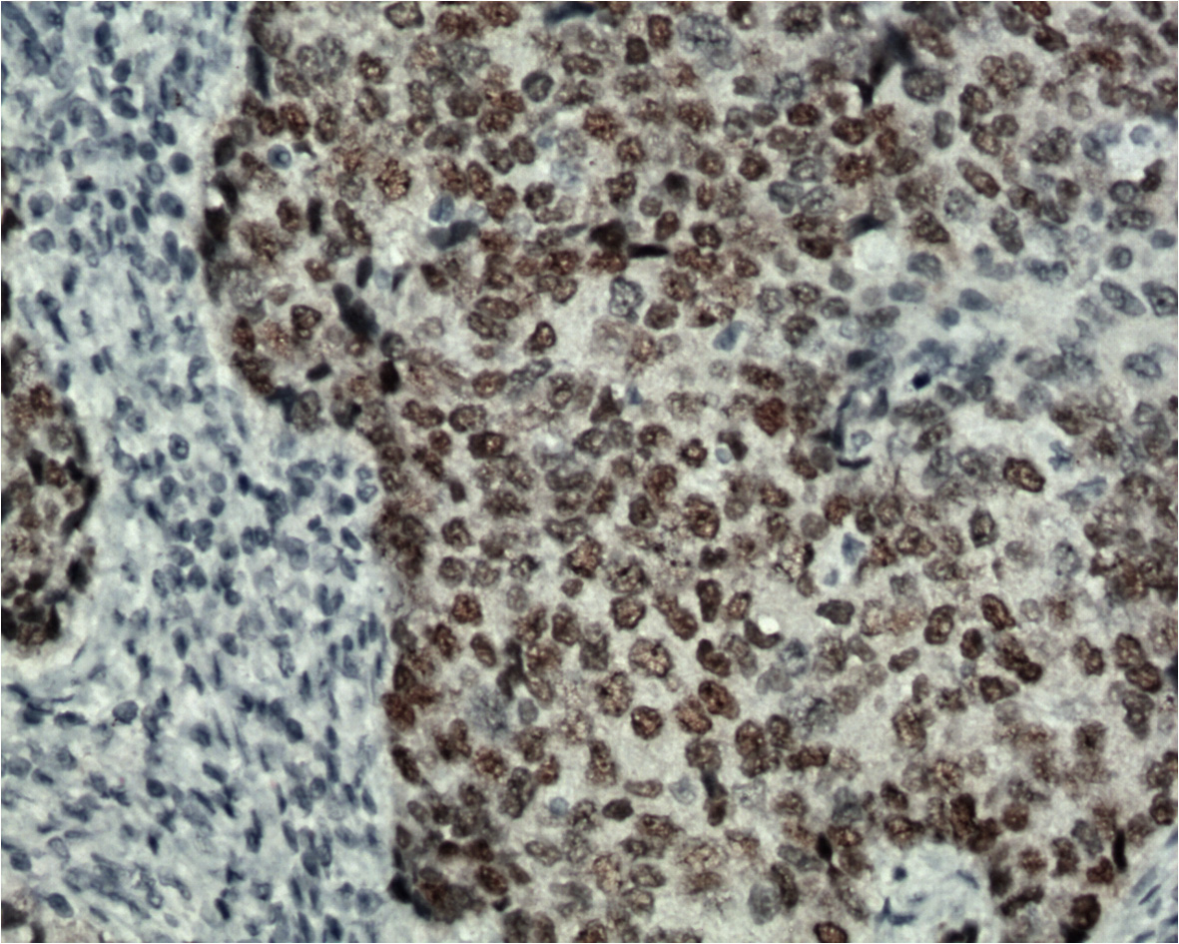
Intense ER immunolabelling in tumor nuclei.

Fine needle aspiration from the lump was suggestive of ductal carcinoma. Genetic analyses could not be conducted for financial/logistics reasons and the diagnosis of NF1 was made based on clinical criteria [3]. Hence a diagnosis of NF1 and breast cancer was made. After staging workup, the patient underwent modified radical mastectomy and axillary lymph node dissection. Histopathological examination revealed invasive ductal carcinoma of Bloom and Richardson histological grade III (Figure 2). The pathological stage was pT3N0M0. On immunohistochemistry, estrogen and progesterone receptors (ER, PgR) were both positive and HER2 was negative (Figure 3).

The patient received 6 cycles of adjuvant FEC (fluorouracil 500 mg/m^2^ q3w, epirubicin 100 mg/m^2^ q3w, cyclophosphamide 500 mg/m^2^ q3w) chemotherapy followed by external beam radiotherapy on a cobalt-60 machine with tangential pairs to chest wall and anterior supraclavicular fields of 50 Gy in 25 fractions with 2 Gy per fraction given once a day for 5 weeks.

At present, the patient is receiving tamoxifen 20 mg once daily and is on follow up since 8 months.

## Discussion

NF1 is an autosomal dominant Mendelian syndrome occurring in about 1 in 4000 persons in the general population and has high penetrance but wide variability in expression [4]. It represents a risk factor for the development of various malignancies, including female breast cancer [5].

**Figure 4:**
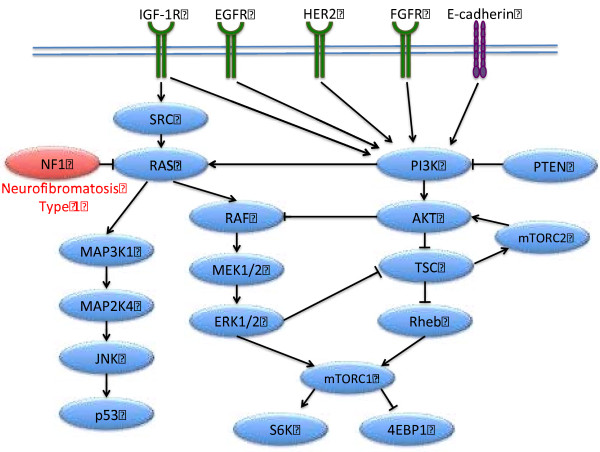
**The *NF1 *** **gene is a negative regulator of Ras, and thus impacts key breast cancer signaling pathways.**

*Neurofibromin 1* (*NF1)* is a tumor suppressor gene that encodes the neurofibromin protein, a negative regulator of the Ras oncogene. *NF1* is located on the pericentromeric region of the long arm of chromosome 17 (which interestingly also houses the *BRCA1* gene), and regulates the conversion of the active Ras-GTP to inactive Ras-GDP (Figure 4) [6]. About 28% of sporadic breast cancers in humans are missing at least one copy of *NF1* gene, either due to deletion or mutation [7].

According to Knudson’s “two-hit” hypothesis, both alleles have to be inactivated for tumorogenesis to occur. In NF1-associated cancers, the first “hit” is a germline mutation, while the second “hit” is a somatic mutation that occurs either *in utero* or after birth and results in inactivation of the normal *NF1* allele and a consequent loss of neurofibromin function. Both alleles of *NF1* need to be inactivated to influence carcinogenesis, but there is emerging evidence that haploinsuficiency or reduced expression may also have a functional impact [8].

Individuals with NF1 are heterozygous for an *NF1* mutation. Mutation in the normal copy of the *NF1* gene enhances the risk of cancer among NF1 patients but these mutations may also be observed in sporadic tumors [9,10].

Women with inherited *NF1* deficiency have an increased risk of breast cancer [11,12]. Some reports implicate spontaneous *NF1* loss in breast tumorigenesis [13,14]. A recent report suggests that females with NF1 have a relative risk of 2.3 (95% confidence interval 1.7 to 2.9) for developing breast cancer compared with a reference cohort [5]. Men also may be at risk (as highlighted by this case report) but there is scarce data on the risk and association of NF1 and male breast cancer due to its rarity [15].

Silencing *NF1* (or other genes including*, BAP1, CDK10, NIPBL, PTEN, RARG, SMC3,* and *UBA3*) have been shown to confer tamoxifen resistance in human breast cancer (MCF7) cell lines [16]. Hence tamoxifen, the drug treatment for hormone receptor positive breast cancers, may theoretically be less effective in cancers involving *NF1* mutations; however, this needs to be corroborated by clinical evidence.

In conclusion, this report aims to describe a rare presentation of a male patient with NF1 and breast cancer. The *NF1* gene is mutated or deleted in a significant proportion of human breast cancers, and its role as a potentially important molecular driver in breast cancers needs to be explored further. One can speculate that drugs blocking the Ras pathway could be useful in breast cancer with *NF1* mutations; this hypothesis needs to be tested in a scientifically robust manner.

## Consent

Informed consent has been obtained from the patient for publication.

## Competing interests

The authors declare that they have no competing interests.
